# Endovascular coil-embolization of an unruptured, true UAA during early pregnancy- a case report

**DOI:** 10.1186/s42155-023-00398-3

**Published:** 2023-10-23

**Authors:** Kai Jannusch, Andrea Steuwe, Lars Schimmöller, Frederic Dietzel, Lena M. Wilms, Daniel Weiss, Farid Ziayee, Tanja Natascha Fehm, Charlotte Schlimgen, Vanessa Poth, Reinhold Thomas Ziegler, Peter Minko

**Affiliations:** 1 Medical Faculty, Department of Diagnostic and Interventional Radiology, University Dusseldorf, Moorenstrasse 5, Dusseldorf, D-40225 Germany; 2 Medical Faculty, Department of Gynecology, University Dusseldorf, Dusseldorf, D-40225 Germany; 3 Department of Gynecology, Hermann-Josef Hospital, Erkelenz, D-41812 Germany; 4 Medical Faculty, Department of Vascular and Endovascular Surgery, University Dusseldorf, Dusseldorf, D-40225 Germany

**Keywords:** Uterine aneurysm, Pregnancy, Coil-embolization

## Abstract

**Background:**

True uterine artery aneurysms, especially during pregnancy, are a rare entity and not well understood. Clinical symptoms are unspecific pelvic pain and pressure. Diagnosis can be confirmed by transvaginal color-coded-sonography and/or magnetic resonance imaging. Because of potential risk of rupture, immediate interdisciplinary discussion and treatment planning in the best interests of both mother and child is crucial.

**Case presentation:**

We present a 31-year-old pregnant woman with increasing pelvic pain and pressure. Diagnosis of an unruptured uterine artery aneurysm was confirmed by color-coded-sonography and magnetic resonance angiography. After interdisciplinary consultation, successful endovascular super-selective coil-embolization was performed by using X-ray fluoroscopy. Thus, fetal radiation dose during treatment with 4.33 mGy (VirtualDoseTM) was as low as possible with no immediate harm to the fetus.

**Conclusions:**

Unruptured true uterine artery aneurysms can be successfully treated by endovascular super-selective coil-embolization during early pregnancy with no immediate harm to the fetus.

## Background

Uterine artery aneurysms (UAA) are an extremely rare medical condition [1–4]. The incidence about true UAA is limited to a few case reports, whereas pseudoaneurysms are the better-known entity [1, 3–5]. While pseudoaneurysms of the uterine artery usually develop after trauma (e.g., after pelvic surgical intervention) the exact genesis of true UAA, especially during pregnancy is unclear [2]. Symptoms include non-specific abdominal and/or pelvic pain, sometimes misinterpreted as renal colic and accompanied by metrorrhagia [5]. They can also be asymptomatic. UAAs can be diagnosed by transvaginal color-coded sonography and Magnetic Resonance Angiography (MRA) or Computed Tomography Angiography (CTA). During pregnancy, transvaginal color-coded sonography should be the first diagnostic tool because of it fast availability and the absence of radiation, dangerous for the fetus. Clear diagnosis and urgent interdisciplinary treatment are important. Due to rarity of this disease, treatment decisions are difficult, because there is still no standard of care. Literature describes an increased risk of rupture if internal iliac artery aneurysms exceed 40 mm (> 6%) [6]. In addition, the guidelines for the treatment of visceral aneurysms of the Society for Vascular Surgery help to find an appropriate treatment decision. Although these guidelines do not provide specific recommendations for uterine artery aneurysms, they suggest the treatment of splenic and renal aneurysms regardless of size during pregnancy. The treatment recommendation lies in the biological rationale that during pregnancy hemodynamic factors and hormonal effects are discussed to weakening the arterial vessel-wall-layers and thus increase risk of aneurysm rupture [7, 8]. This rationale can be applied to all aneurysms during pregnancy regardless of the specific localization. Treatment has evolved in recent years from open surgical treatment to less invasive image-guided interventions. Latter are associated with a decrease in morbidity and mortality, especially during pregnancy, which strengthen the choice as first-line therapy. Nevertheless, the image based treatment causes a divergent risk of radiation-related adverse events for the embryo and fetus according to gestational age. Thus, treatment should be performed strict according to predefined guidelines of X-ray guided procedures in pregnancy [9]. In this case report we present a rare case of a true UAA during pregnancy with successful coil-embolization.

## Case presentation

**Fig. 1:**
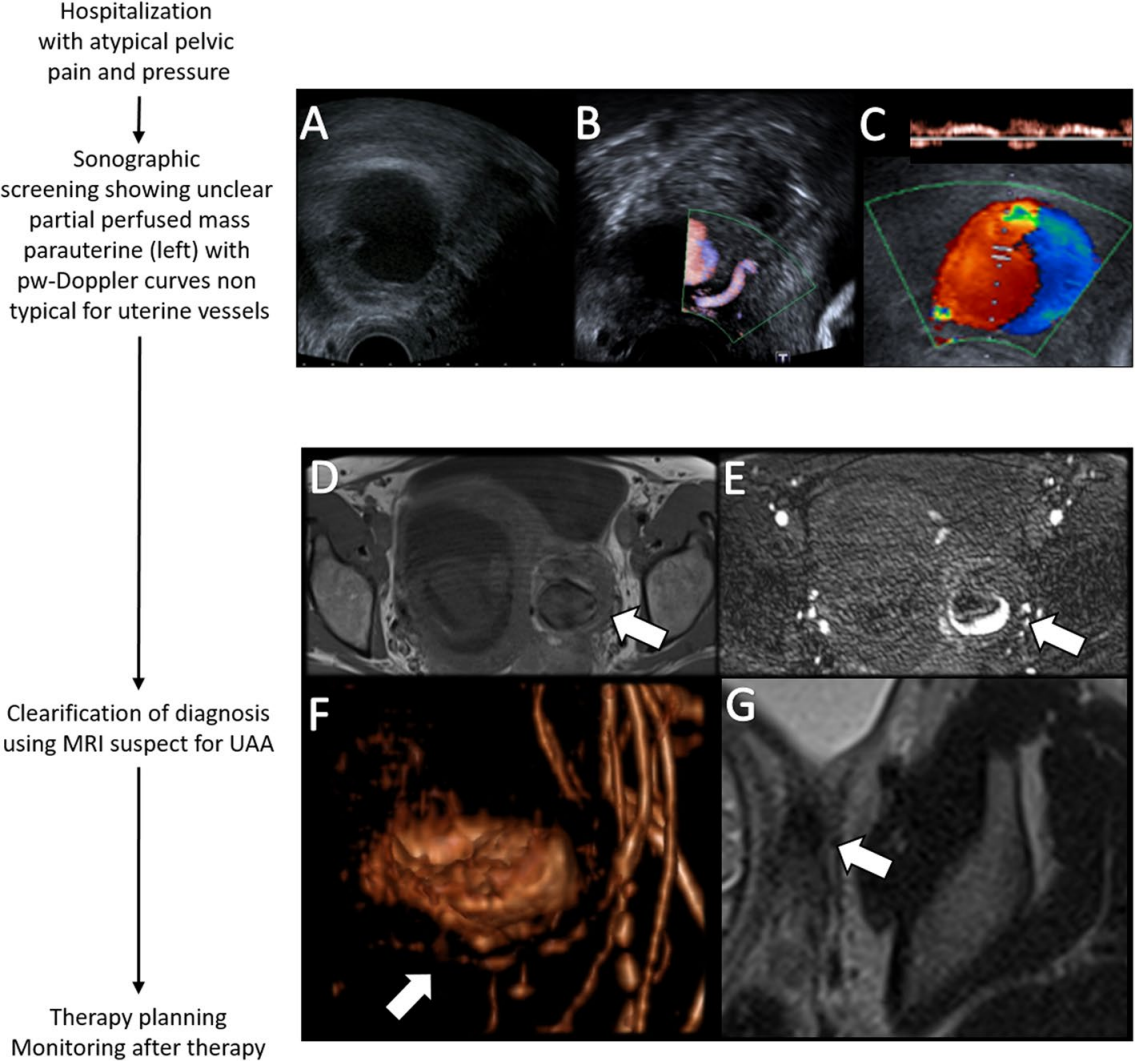
Pregnant women presented with untypical pelvic pain and pressure showing a hypoechoic parauterine mass at B-Mode sonography measuring 39 mm (**A**). Positional relation to the uterine artery (**B**) and intralesional flow at color-coded sonography with pw-Doppler curves non typical for uterine vessels and typical yin-yang sign for an aneurysmatic lesion (**C**). Clarification of diagnosis suspect for UAA using contrast enhanced MRI (**D**-**F**). Contrast enhanced QUISS-sequence showing intra-aneurysmatic collection of contrast agent (**E**, arrow) comparing to native T1w-sequence (**D**, arrow). MR angiography shows the exact anatomy of the internal iliac artery with the true aneurysm originating from the uterine artery (**F**, arrow). Control MRI before the calculated date of birth showed a significant decreased and still occluded aneurysm (**G**, arrow)

A 31-year-old patient (first gravida, 20 + 4 weeks of gestation) presented to the emergency department of an external hospital with sudden left-sided pelvic pain and pressure. Physical examination was unremarkable, but transvaginal sonography and MRI revealed an unknown left parauterine mass with a maximum diameter of 39 mm (Fig. 1A). The patient was transferred to a specialized center with high diagnostic and interventional expertise for clarification of diagnosis and further treatment. Blood count showed slightly elevated infection parameters and slightly reduced but stable hemoglobin at 9.4 g/dl (norm value: 11.9 to 14.6 g/dl). Cardiotocogram and fetal sonography were unremarkable, while intensified transvaginal color-coded sonography including pulse-waved-Doppler showed a perfusion of the parauterine mass and yin-yang sing, typical for an aneurysmatic lesion (Fig. 1B, C). Additional pelvic MRI with MR angiography led to the diagnosis of a partially thrombosed true aneurysm originating from the left uterine artery (Fig. 1D-F). There were no signs of rupture (i.e., intra-abdominal free fluid) but the UAA showed an increase in size of 7 mm within 6 days. Due to the increasing risk of rupture, an immediate interdisciplinary therapy-discussion was initiated including feto-maternal specialists, gynecologists, an interventional radiologist and a vascular surgeon. All concluded that based on the early gestational age, the size progression of the true UAA and considering the persistent pain, selective embolization should be the treatment of choice. Afterwards the patient was intensively informed about procedural risks associated with the treatment in relation to her pregnancy and signed specified informed consent form.

**Fig. 2:**
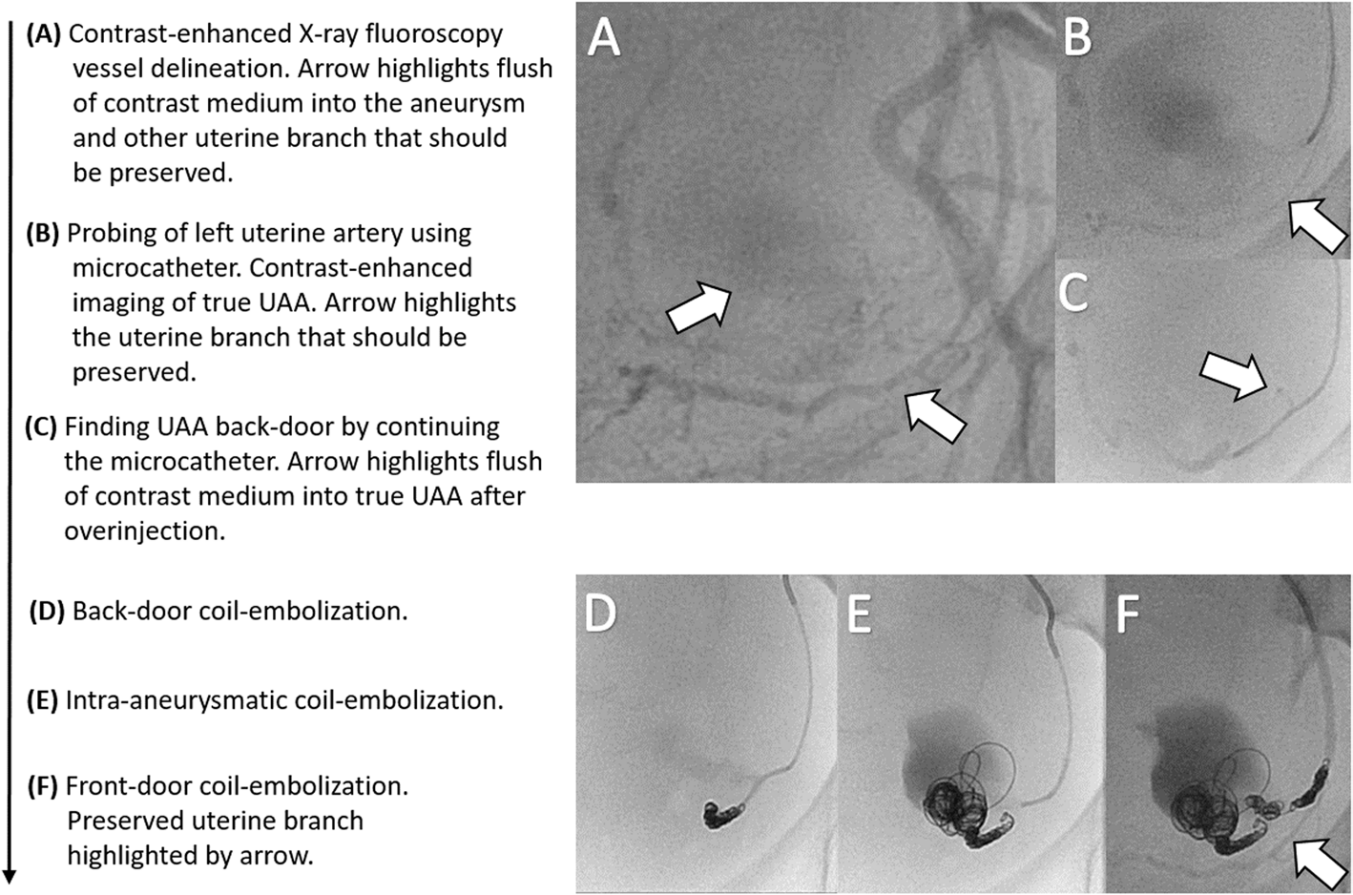
Endovascular contrast enhanced vessel delineation using X-ray fluoroscopy instead of DSA with flush of contrast medium into the aneurysm (**A**). After selective probing of the left uterine artery with a microcatheter better visualization of the true UAA and demarcation of another uterine branch (arrow) that should be preserved during embolization (**B**). Back-door of the true UAA and a little flush of contrast medium (arrow) after overinjection (**C**). Back-door (**D**), intra-aneurysm (**E**) and front-door (**F**) coil-embolization preserving the initial shown other uterine branch (**F**, arrow). In the final X-ray fluoroscopy, there is no contrast enhancement of the uterine vessel leading to the UAA (**F**). Altogether 34 coils (Cook: 2 mm Hilal, 3 mm / 8 mm / 10 mm Nester; BostonScientific: 3 mm IDC, 20 mm Interlock) were used for embolization

**Fig. 3:**
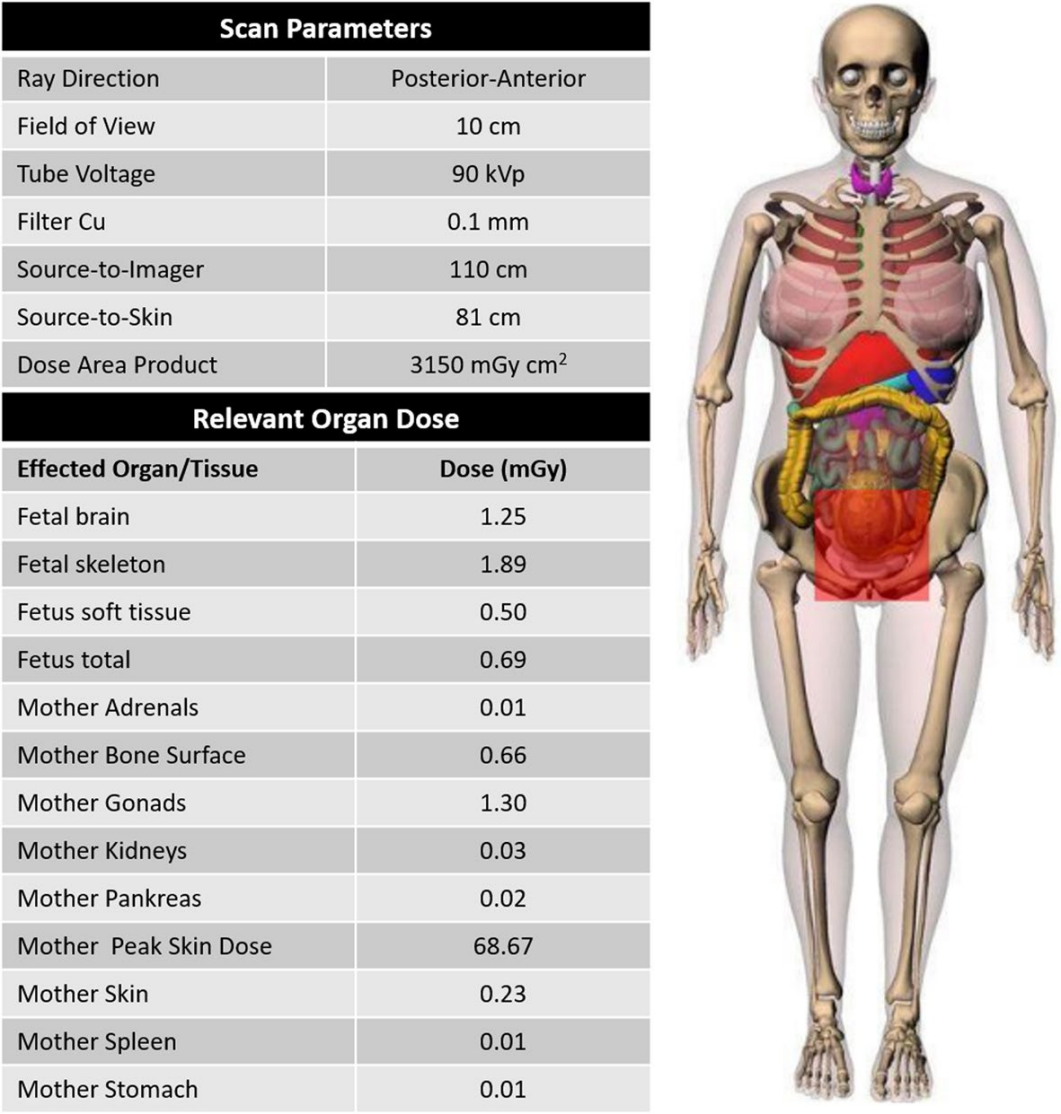
Results of model based radiation dose calculation during coil-embolization using VirtualDose™ with a total fetal dose of 4.33 mGy. Limitations of the calculation are given by the model calculation itself, the limited fitting field size and limited fitting filters

The intervention taking 79 min was performed under local anesthesia with retrograde access via the right common femoral artery. Contrast agent application under X-ray fluoroscopy secured the diagnosis of a true left UAA (Fig. 2A-C). Embolization was performed by super-selective back-door, intra-aneurysm and front-door coil-embolization via a 2.7 F microcatheter (Progreat, Terumo) with a total of 34 coils (Cook: 2 mm Hilal, 3 mm / 8 mm / 10 mm Nester; BostonScientific: 3 mm IDC, 20 mm Interlock; see also Fig. 2D-F). Due to superselectivity, a more proximally originating small vascular branch of the left uterine artery could be preserved to ensure continued left-sided supply of the uterus (Fig. 2F). Control X-ray fluoroscopy showed complete occlusion of the aneurysm (Fig. 2F). A total amount of 25 ml Accupaque 300 was applied. Fluoroscopy time was 13 min. The model calculated radiation dose to the fetus using VirtualDose^™^ software was 4.33 mGy (Fig. 3). Follow-up sonographic examination 15 days post-treatment confirmed a healthy mother, fetus, and a still eliminated true UAA. The pregnancy continued without complications and a control MRI before the calculated date of birth to plan the birth process showed a significant decreased and still occluded aneurysm (Fig. 1G), so that a regular childbirth was possible. Finally, a healthy child was born without the need for anticipation of birth.

## Conclusions

This case is an example of a rare diagnosed, unruptured true UAA during pregnancy and highlights the importance of a well-cooperating interdisciplinary team. Diagnosis is challenging, especially with regard to nonspecific clinical symptoms or asymptomatic patients. Lower abdominal- or back-pain and sensation of pressure as one of the main symptoms may give a hint to the condition [1, 3, 5]. Due to risk of rupture quick diagnosis and treatment is crucial [6, 10]. Treatment has evolved over the past decades moving away from surgical procedures including laparotomy and internal iliac artery ligation [11]. Due to reduced mortality and morbidity, actually image guided inventions are the treatment of choice in hemodynamic stable patients, consisting for e.g. of coil embolization, stent implantation or thrombin injection [12–14]. Image guided inventions carry the risk of deterministic or stochastic effects to the embryo and fetus depending on the gestational age. Thus, during pregnancy, minimization of radiation exposure is from extreme importance. The first 14 days post-conception bearing the highest risk of adverse embryonal effects including death (“all or none” phenomenon) when reaching a radiation dose of 150–200 mGy. The radiosensitivity decreases as the embryo grows. Reaching the third trimester, there is the lowest risk for malformation and mental retardation. In general, a radiation dose below 50 mGy poses no evidence of an elevated risk of fetal anomalies, intellectual disability, growth retardation or pregnancy loss [15]. Nonetheless, high interventional expertise and adherence to the ALARA principle is important [9]. Thus, during angiography, the visualization and therapy control of the UAA should be performed only by X-ray fluoroscopy instead of digital subtraction angiography as presented in our case. Therefore, pre-procedural planning using MR-Angiography was of critical importance in this case and radiation exposure can be significantly reduced by modifying the exposure time, the number of images obtained, beam size, and imaging area. There were no diagnostic or therapeutic disadvantages from the use of X-ray fluoroscopy in our case and the administered dose is not associated with further deterministic (non-stochastic) fetal effects (4.33 mGy; see Fig. 3) because the critical threshold of 50 mGy was not reached [16]. Postnatal hypothyroidism of the newborn is also discussed as potential risk after image based intervention using iodinated contrast agents during pregnancy. The underlying idea is that the newborn has not the ability to fully escape from the acute Wolff–Chaikoff effect until approximately 36 weeks [17]. However, until now this has not been demonstrated in the existing literature [17, 18]. Nonetheless, the amount of administered iodinated contrast agents should be kept low during an interventional procedure of pregnant patients and the thyroid function should be checked in the first few days of life of the newborn [9]. For our patient during early pregnancy, super-selective coil-embolization was the treatment of choice because it has the highest success rate and the lowest risk for the fetus. Only 2D coils were placed during the procedure because the aneurysm should not be coiled completely. The main goal was to stop the inflowing blood, stimulate the thrombogenicity to achieve occlusion of the UAA and furthermore maintain physiological space conditions in order to avoid a possible increased pressure on the fetus during further growth due to too many placed coils. Thus, due to good collateralization of the uterine artery, the proximal and distal sites of the UAA must be occluded, also known as front-/back-door embolization. As presented by our case and other reports in the literature uterine blood flow can be sacrificed safely in a potentially life threatening condition. Blood supply from collaterals and the contralateral uterine artery allowed the pregnancy to continue safely [19].

Consequently, a coil embolization of experienced radiologists is an effective and feasible treatment for the pregnant women and furthermore safe for the fetus.

## Data Availability

The datasets used and/or analysed during the current study are available from the corresponding author on reasonable request.

## Abbreviations

UAA: Uterine artery aneurysms
MRA: Magnetic Resonance Angiography
CTA: Computed tomography angiography
